# Combined Kinetin and Spermidine Treatments Ameliorate Growth and Photosynthetic Inhibition in *Vigna angularis* by Up-Regulating Antioxidant and Nitrogen Metabolism under Cadmium Stress

**DOI:** 10.3390/biom10010147

**Published:** 2020-01-16

**Authors:** Mohammad Abass Ahanger, Usman Aziz, Abdulaziz Abdullah Alsahli, Mohammed Nasser Alyemeni, Parvaiz Ahmad

**Affiliations:** 1 College of Life Sciences, Northwest A&F University, Yangling 712100, China; ahangerma@gmail.com; 2 College of Agronomy, Northwest A&F University, Yangling 712100, China; usmanaziz@gmail.com; 3 Botany and Microbiology Department, College of Science, King Saudi University, P.O. Box. 2460, Riyadh 11451, Saudi Arabia; aalshenaifi@ksu.edu.sa (A.A.A.);; 4 Department of Botany, Sri Pratap College, Srinagar 190001, Jammu and Kashmir, India

**Keywords:** oxidative damage, antioxidants, secondary metabolites, osmolytes, cadmium, *Vigna angularis*

## Abstract

Pot experiments were conducted to investigate the probable beneficial role of the individual as well as combined application of kinetin (50 μM Kn) and spermidine (200 μM Spd) on *Vigna angularis* under cadmium (Cd) stress. Cd treatment reduced growth by declining the content of chlorophylls and carotenoids, photosynthesis, and gas exchange parameters. Exogenously, Kn and Spd application enhanced the photosynthetic parameters and up-regulated the antioxidant system by improving the activities of antioxidant enzymes and the content of non-enzymatic components. In addition, the application of Kn and Spd resulted in significant improvement in the content of sugars, proline, and glycine betaine, ameliorating the decline in relative water content. Oxidative stress parameters including hydrogen peroxide, superoxide, lipid peroxidation, lipoxygenase activity, and electrolyte leakage increased due to Cd stress; however, the application of Kn and Spd imparted a significant decline in all these parameters. Further, reduced Cd uptake was also observed due to Kn and Spd application. Total phenols and flavonoids also increased due to Kn and Spd treatments under normal as well as Cd stress conditions, which may have further helped with the elimination of reactive oxygen species. Reduction in the activity of nitrate reductase and the content of nitrogen was ameliorated due to the exogenous application of Kn and Spd. Therefore, the exogenous application of Kn and Spd benefited *Vigna angularis* counteracting the damaging effects of Cd stress by up-regulating the tolerance mechanisms, including antioxidant and osmolyte metabolism.

## 1. Introduction

Plants are sessile and continuously exposed to several environmental threats, resulting in a considerable reduction in growth and yield. Among the key concerns are included the heavy metal(loid) pollution of agricultural soils and faulty irrigation systems. Once entering into the food chain through the plant system, heavy metals can affect the human population by damaging different organs. Among the toxic heavy metals, cadmium (Cd) is added to soil from natural and anthropogenic sources, thereby affecting the existing flora [1]. Cd shows high mobility in the soil–plant system and has been reported to inhibit photosynthesis by down-regulating the chlorophyll and Rubisco synthesis, restricting the electron transport by increasing reactive oxygen species (ROS) production [2], and affecting the functioning of Calvin cycle enzymes [3,4]. The damaging effects of Cd at physiological and biochemical levels have been largely attributed to the excessive generation of ROS resulting in protein degradation, nucleic acid damage, photoinhibition, and membrane damage [5,6,7]. Indigenously occurring tolerance mechanisms that include the antioxidant system, the accumulation of compatible osmolytes, and secondary metabolites are employed to counteract the damaging effects of stresses [6,8]. The antioxidant system is comprised of enzymatic and non-enzymatic components [9,10]. Efficient working of the antioxidant system, osmolyte, and secondary metabolite metabolism results in the quick elimination of ROS from different cellular components for better stress amelioration and growth maintenance [2,10]. The accumulation of Cd in edible plant parts degrades yield quality and results in serious health hazards to humans and other animals.

Plant growth hormones mediate stress signaling, assisting actively in the mitigation of adverse effects of stress factors [8,11,12]. Cytokinins (CKs) include a class of low-molecular weight compounds that have crucial roles in plant growth and development [13]. CKs regulate cell division and morphogenesis, apical dominance, seed germination, flower and seed development, the uptake of nutrients, assimilate into sink organs, and delay senescence. CK initiates a signal transduction cascade by binding to membrane-bound receptors, therefore activating the primary hormone response and several genes affecting the synthesis, transport, metabolism, and perception of CKs in plants [14,15,16]. Kinetin (Kn) is a synthesized cytokinin that improves plant growth under extreme conditions including water logging [17], salinity [8,18], and Cd [7] stress conditions. The exogenous application of CK (6-benzyladenine) prevents ROS-mediated photoinhibition by up-regulating the antioxidant system [19]. Cytokinin overproduction improves drought stress tolerance by enhancing the metabolite accumulation [20]. Polyamines (PAs) are a group of low molecular weight nitrogenous compounds that have been reported to mediate several plant development events including photosynthetic regulation under stressful conditions [21]. Among the common occurring PAs are diamine putrescine, triamine spermidine, and tetra-amine spermine, existing ubiquitously in plants and playing a crucial role in plant developmental processes [22,23]. Due to their polycationic nature, PAs can interact with proteins, phospholipids, nucleic acids, and cell wall components, leading to their stabilization at physiological pH [22]. The differential influence of stresses on the PA metabolism has been reported [24,25], and their involvement in key plant processes such as membrane stabilization, ROS scavenging, protein synthesis, enzyme activation, mineral uptake, and hormonal profile regulation has been reported (reviewed by Ahmad et al. [22], Hu et al. [24], Puyang et al. [25], and Li et al. [26]). However, possible antagonistic or synergistic interaction between PA and Kn has not been worked, therefore prompting us to undertake the current investigation.

*Vigna angularis,* commonly known as mung bean or adzuki bean, is widely grown for its small beans throughout the world. It forms an important part of human food because of its rich content of carbohydrates, proteins, and dietary fiber. Throughout the world, heavy metal pollution has been one of the prime factors for its declined yield, which can also seriously influence animal health, including that of humans. Therefore, in present study, we hypothesized that the exogenous application of Kn and Spd application can protect adzuki beans from the damaging effects of Cd by up-regulating the tolerance mechanisms, including the antioxidant system and osmolyte metabolism.

## 2. Materials and Methods

Healthy seeds of *Vigna angularis* were sterilized using 5% NaOCl for 5 min followed by repeated washing with distilled water. Thereafter, seeds were sown in earthen pots filled with sand, peat, and perlite in the ratio of 2:1:1. At the time of sowing, the pots received 200 mL of full strength Hoagland solution. Ten days after germination, three seedlings per pot were maintained and grown for further experimentation. Pots were divided into different groups and were foliarly supplied with either kinetin (6-furfurylaminopurine, Kn, 50 μM) or spermidine (Spd, 200 μM) or Kn + Spd twice a week for another 20 days. Cadmium stress was initiated by applying 100 μM of Cd (as CdCl_2_). Control plants were sprayed with an equal volume of distilled water. Pots were maintained in a greenhouse and were regularly monitored. After 30 days of seedling growth i.e., 20 days after Kn, Spd, and Cd treatment, plants were carefully uprooted and were separated into leaves, stems, and roots. Height was measured by a manual scale, while for dry weight estimation, whole seedlings were oven dried. Cd content was estimated in oven-dried shoots and roots. For the estimation of photosynthesis, oxidative stress parameters, and activities of enzymes, fresh leaves were used. Different parameters studied are described below.

### 2.1. Estimation of Photosynthetic Pigments, Photosynthesis, and Relative Water Content (RWC)

Photosynthetic pigments from fresh leaf tissues were extracted in acetone (80%), and the optical density of the supernatant was measured spectrophotometrically at 480, 645, and 663 nm [27].

For the determination of net photosynthetic rate (*Pn*), intercellular CO_2_ concentration (Ci), and transpiration rate (*E*), fully expanded upper leaves were used, and observations were recorded using a portable photosynthetic system (LICOR, LI-6400).

RWC was estimated following Smart and Bingham [28], using the following formula:

$$\mathrm{RWC}=\frac{\mathrm{Fresh}\;\mathrm{weight}\;-\;\mathrm{Dry}\;\mathrm{weight}}{\mathrm{Turgid}\;\mathrm{weight}\;-\;\mathrm{Dry}\;\mathrm{weight}}\times \;100.$$

### 2.2. Estimation of Electrolyte Leakage, Hydrogen Peroxide (H_2_O_2_) and Superoxide (O_2_^–^)

The method of Dionisio-Sese and Tobita [29] was adopted for determining electrolyte leakage using the following formula:

Percent electrolyte leakage = (EC1 − EC0)/(EC2 − EC0) × 100

Hydrogen peroxide (H_2_O_2_) in fresh leaf tissues was estimated by a spectrophotometric method, and absorbance was measured at 390 nm [30]. The concentration of H_2_O_2_ was determined from a standard curve and expressed as nmol g^−1^ FW.

For measurements of O_2_^−^ concentrations, fresh tissue was homogenized in potassium phosphate buffer (65 mM, pH 7.8), and homogenate was centrifuged at 5000× *g*. Supernatant was mixed with 10 mM of hydroxylamine hydrochloride and left for 20 min followed by the addition of sulfanilamide and naphthylamine. After 20 min of incubation at 25 °C, absorbance was measured at 530 nm [31], and calculations were done using the standard curve of NaNO_2_.

### 2.3. Lipid Peroxidation and Lipoxygenase Activity

Lipid peroxidation was determined by measuring the formation of malondialdehyde content, as described by Madhava Rao and Sresty [32]. An extinction coefficient of 155 mM^−1^cm^−1^ was used for calculation.

The activity of lipoxygenase (LOX; EC 1.13.11.12) was assayed following the method of Doderer et al. [33]. Change in absorbance was monitored at 234 nm using linoleic acid as a substrate, an extinction coefficient of 25 mM^−1^ cm^−1^ was used for calculation, and activity was expressed as units mg^−1^ protein.

### 2.4. Estimation of Nitrate Reductase Activity

For assaying nitrate reductase (NR; EC 1.6.6.1) activity, 300 mg of fresh leaf tissue was incubated in 100 mM of potassium phosphate buffer (pH 7.5) containing 200 mM of KNO_3_ and 0.5% *n*-propanol (*v*/*v*) at 30 °C for 3 h in dark. Thereafter, an aliquot (1 mL) was mixed with an equal volume of 1% sulphanilamide and 0.2% 1-nephthylethylene diamine dihydrochloride, and the mixture was allowed to stand for 20 min. Thereafter, the absorbance was recorded at 540 nm [34].

### 2.5. Estimation of Proline, Glycine Betaine, and Sugars

For the estimation of proline in the leaf tissue, the method described by Bates et al. [35] was followed. Proline was extracted with toluene, and the absorbance was read at 520 nm. A standard curve of proline was used for the determination of concentrations, which were expressed as µmol proline g^−1^ fresh weight.

The method described by Grieve and Grattan [36] was adopted for the estimation of glycine betaine and the formation of periodide crystals was read at 365 nm after reaction with cold KI–I_2_ reagent under acidic conditions. Concentrations were determined from a standard curve of glycine betaine.

The sugar content in dried leaf samples was extracted in ethanol, and the extract was centrifuged at 5000× *g* for 20 min. The supernatant was mixed with anthrone reagent, and absorbance was taken at 620 nm as described by Ahanger et al. [37].

### 2.6. Determination of Antioxidant Enzyme Activities

Enzymes were extracted from fresh leaf tissue (0.5 gm) in ice-cold potassium phosphate buffer (100 mM, pH = 7.0) containing polyvinylpyrrolidone (PVP, 1%) using a prechilled mortar and pestle. The homogenate was centrifuged at 12,000× *g* for 15 min at 4 °C, and the supernatant was used as an enzyme source for assay.

For measuring the activity of superoxide dismutase (SOD, EC 1.15.1.1) the ability of the enzyme extract to inhibit the photochemical reduction of nitroblue tetrazolium (NBT) was measured at 560 nm. An assay mixture containing 100 mM of phosphate buffer (pH 7.4), 10 mM of methionine, 1.0 mM of Ethylenediaminetetraacetic acid (EDTA), 50 µM of riboflavin, 75 µM of NBT, and 100 µL of enzyme extract was incubated for 15 min under fluorescent light. The optical density was measured at 560 nm, and the activity was expressed as U mg^−1^ protein [38].

Catalase (CAT, EC 1.11.1.6) activity was assayed by monitoring the decomposition of H_2_O_2_ at 240 nm for 2 min. The reaction mixture contained 50 mM of phosphate buffer (pH 6.0), 0.1 mM of EDTA, 20 mM of H_2_O_2_, and 100 µL of enzyme extract in a final volume of 2 mL. The activity was expressed as U mg^−1^ protein, and an extinction coefficient of 39.4 mM^−1^cm^−1^ was used [39].

The method of Nakano and Asada [40] was employed for measuring the activity of ascorbate peroxidase (APX, EC 1.11.1.1), and change in absorbance was recorded at 290 nm for 2 min. The reaction mixture (2 mL) contained 50 mM of phosphate buffer (pH 7.5), 0.1 mM of EDTA, 0.3 mM of ascorbate, and 100 µL of enzyme extract, and the reaction was initiated by adding 100 µL of H_2_O_2_. A molar extinction coefficient of 2.8 mM^−1^ cm^−1^ was used for the calculation of APX activity, which was expressed as U mg^−1^protein.

Glutathione reductase (GR, EC1.6.4.2) was assayed by measuring the change in absorbance at 340 nm for 3 min. The reaction mixture contained 100 mM of potassium phosphate buffer (pH 7.0), 1.0 mM of EDTA, 50 μM of NADPH, 100 μM of oxidized glutathione, and 100 μL of enzyme in a final volume of 3 mL, and the activity was expressed as U mg^−1^ protein [41].

The method of Nakano and Asada [40] was employed for the estimation of dehydroascorbate reductase (EC 1.8.5.1) (DHAR) activity by monitoring the change in absorbance at 265 nm for 1 min. For calculation extinction, a coefficient of 14 mM^−1^ cm^−1^ was used.

Glutathione *S*-transferase (GST, EC: 2.5.1.18) was estimated according to the procedure of Hasanuzzaman and Fujita [42], and change in absorbance was read spectrophotometrically at 340 nm for 2 min. The activity of GST was calculated using the extinction coefficient of 9.6 mM^−1^ cm^−1^.

### 2.7. Estimation of Ascorbate, Reduced Glutathione, and Tocopherol

Ascorbate (AsA) content was determined by macerating fresh plant material in 6% trichloroacetic acid (TCA), and the supernatant was mixed with 2% dinitrophenylhydrazine and 10% thiourea. After incubating in a water bath for 15 min, samples were cooled, and 5 mL of cooled 80% H_2_SO_4_ was added. Absorbance was taken at 530 nm [43]. The standard curve of AsA was used for calculation.

The estimation of reduced glutathione (GSH) was carried by following method described by Ellman [44]. First, 100 mg of fresh tissue was homogenized in phosphate buffer (pH 8.0), and 500 µL of supernatant was mixed with 5,5-dithiobis-2-nitrobenzoic acid. Optical density was read at 412 nm, and calculation was done from a standard curve of GSH.

Tocopherol was extracted in ethanol and petroleum ether (1.6:2). After centrifugation, 1 mL of supernatant was incubated with 2% of 2,2-dipyridyl in dark followed by the addition of distilled water (4 mL). Absorbance was recorded at 520 nm [45], and calculation was done from a standard curve.

### 2.8. Total Phenols and Flavonoids

Total phenolic content was determined by Folin–Ciocalteu reagent using the method of Malick and Singh [46] and expressed as mg gallic acid equivalent (GAE) g^−1^ of extract (mg g^−1^). The method of Zhishen et al. [47] was employed for the estimation of flavonoid content. To 0.5 mL of plant extract was added 0.150 mL of sodium nitrate (5%), which was left for five minutes followed by the addition of 10% aluminum chloride and 1 M of sodium hydroxide. After 15 min, absorbance was recorded at 510 nm.

### 2.9. Estimation of K, N, and Cd

K were estimated flame photometrically, as described by Ahanger et al. [37]. Micro-Kzeldahl’s method as suggested by Jackson [48] was employed for the estimation of N content. For the determination of Cd, a dry 500 mg plant sample was acid digested in sulfuric acid and nitric acid (1:5, *v*/*v*) at 60 °C for 24 h. Thereafter, the concentration of Cd in digested samples was estimated using atomic absorption spectrophotometer [5].

### 2.10. Statistical Analysis

Data presented is the means of four replicates with ± SE calculated. The data was subjected to statistical analysis using analysis of variance (ANOVA) for a completely randomized design and least significant difference (LSD) calculated at 0.05%.

## 3. Results

### 3.1. Kn and Spd Application Reduced the Cd Uptake and Improved Growth

The treatment of Cd reduced the plant height and dry biomass significantly over the control and exogenous Kn and Spd individually as well as combinedly, which assuaged the decline to a considerable extent. Relative to control, the height and dry weight was reduced by 31% and 35% respectively due to Cd, while a maximal amelioration of 33% and 37% was observed due to Kn + Spd treatment over the Cd-stressed seedlings (Table 1). Cd accumulation was reduced by 39%, 36%, and 61% in the shoots and by 20%, 27%, and 37% in the roots due to Kn, Spd, and Kn + Spd treatment respectively over the Cd-stressed plants (Table 1).

### 3.2. Application of Kn and Spd Improves Pigment Synthesis and Photosynthesis

The exogenous application of Kn and Spd significantly improved the synthesis of chlorophylls and carotenoids, attaining maximal values with their combined application. Relative to control, total chlorophylls increased by 28% and total carotenoids increased by 30% due to Kn + Spd application. Cd stress resulted in a decline of 31% for total chlorophylls, 37% for carotenoids, 44% for net photosynthesis, 35% for transpiration, and 32% for intercellular CO_2_ concentration (Table 1). The application of Kn and Spd individually ameliorated the Cd-mediated decline in pigments and gas exchange parameters considerably; however, maximal amelioration rates of 33%, 35%, 45%, 35%, and 37% for total chlorophylls, carotenoids, net photosynthesis, transpiration, and intercellular CO_2_ concentration over Cd-stressed plants was observed due to Kn and Spd application (Table 1).

### 3.3. Kn and Spd Application Reduced Cd-Induced Oxidative Stress

**Figure 1:**
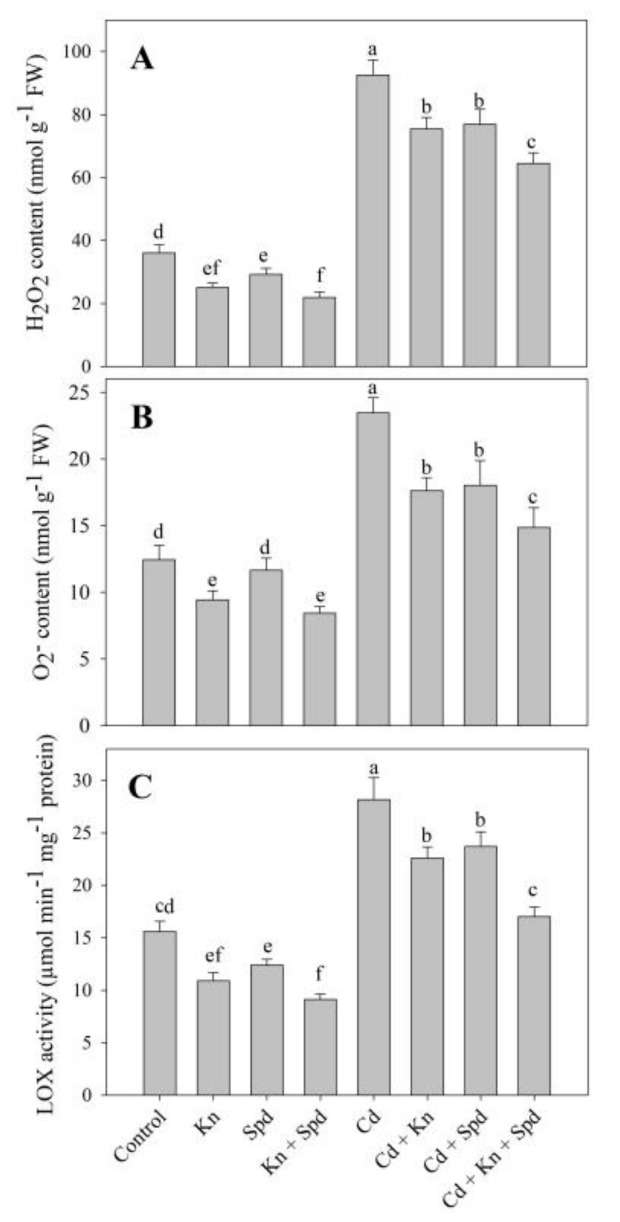
Effect of cadmium stress on (**A**) hydrogen peroxide, (**B**) superoxide, and (**C**) lipoxygenase activity in *Vigna angularis* seedlings treated with exogenous kinetin (Kn), superoxide dismutase (Spd), or Kn + Spd application. Data is mean (± SE) of four replicates and different letters represent significant difference at *p* < 0.05.

**Figure 2:**
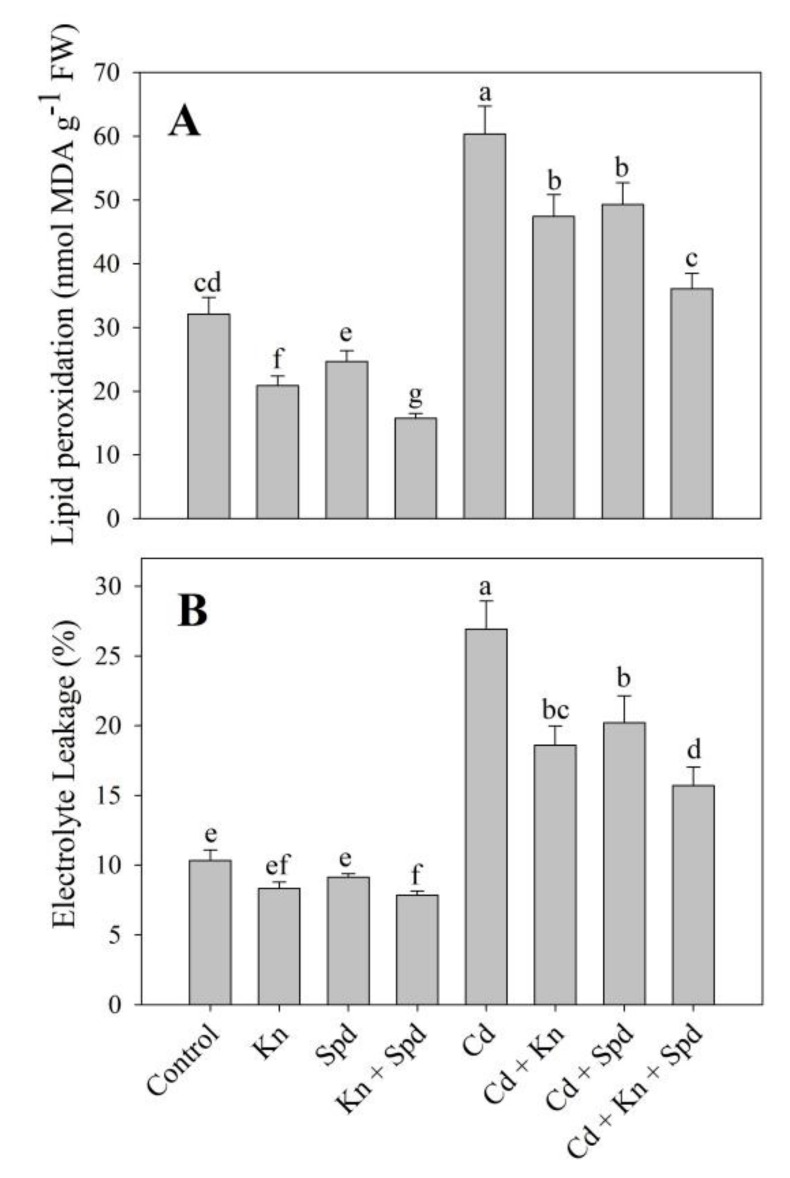
Effect of cadmium stress on (**A**) lipid peroxidation, and (**B**) electrolyte leakage in *Vigna angularis* seedlings treated with exogenous Kn, Spd, or Kn + Spd application. Data is mean (±SE) of four replicates and different letters represent significant difference at *p* < 0.05.

The application of Kn and Spd reduced the generation of ROS including H_2_O_2_ and O_2_, thereby lessening the lipid peroxidation and electrolyte leakage (EL). Cd-stressed seedlings exhibited an increased accumulation of H_2_O_2_ (61%) and O_2_^−^ (47%) (Figure 1A,B), resulting in a 46% increase in lipid peroxidation and a 61% increase in electrolyte leakage (Figure 2A,B).However, the application of Kn and Spd imparted an apparent effect by declining their generation and preventing the membrane damage. Relative to Cd, the combined application of Kn + Spd reduced the Cd-induced accumulation of H_2_O_2_ and O_2_^−^ maximally by 30% and 36%, resulting in 40% and 41% amelioration of the lipid peroxidation and electrolyte leakage (Figure 1A,B and Figure 2A,B). In addition, Kn and Spd application reduced lipoxygenase activity by 30% and 20%, attaining a maximal decline of 41% with Kn + Spd application over the control. Cd stress triggered the activity of lipoxygenase by 44%, which was declined by the application of Kn and Spd with a maximal decline of 39% in Cd + Kn + Spd treatment (Figure 1C).

### 3.4. Effect of Kn and Spd on Accumulation of Sugars, Proline, and Glycine Betaine, and RWC

**Figure 3:**
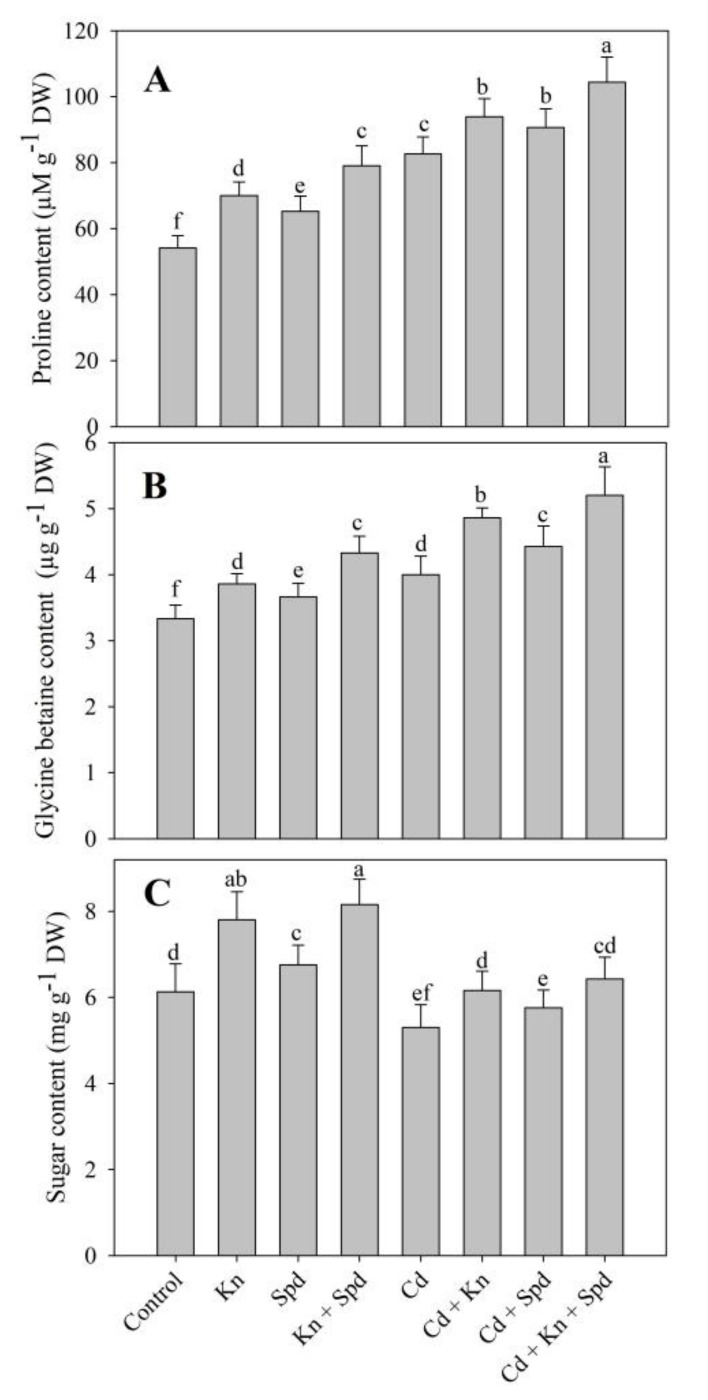
Effect of cadmium stress on (**A**) proline, (**B**) glycine betaine, and (**C**) sugar content in *Vigna angularis* seedlings treated with exogenous Kn, Spd, or Kn + Spd application. Data is the mean (±SE) of four replicates, and different letters represent a significant difference at *p* < 0.05.

Proline (34%) and glycine betaine (GB) (16%) increased due to Cd stress; however, sugar (13%) content declined over the control plants. The application of Kn and Spd alone and combined increased the accumulation of proline, GB, and sugars under normal and Cd stress conditions. Individually, Kn was much more effective compared to Spd. Relative to control, the percent increase in proline, glycine betaine, and sugars was 31%, 23%, and 24% respectively in Kn + Spd-treated seedlings and caused further enhancement in the accumulation of proline and glycine betaine under Cd stress, reaching 48% and 35% over control. However, the Cd-induced decline in sugar content was ameliorated completely due to the combined application of Kn and Spd (Figure 3A–C). Relative to control, RWC declined by 30% due to Cd stress and an amelioration of 18%, 14% and 22% was observed due to Kn, Spd, and Kn + Spd application (Table 1).

### 3.5. Kn and Spd Application Influences the Antioxidant System

**Figure 4:**
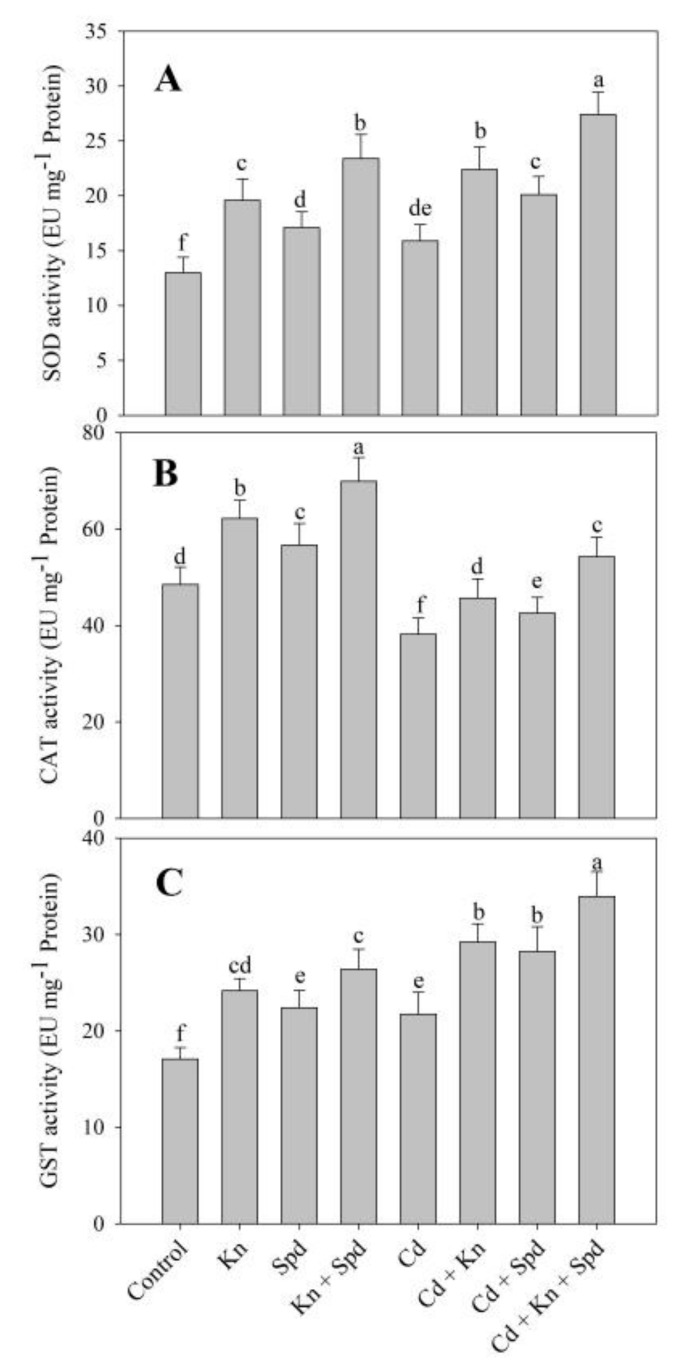
Effect of cadmium stress on the activity of (**A**) superoxide dismutase, (**B**) catalase, and (**C**) glutathione *S*-transferase in *Vigna angularis* seedlings treated with exogenous Kn, Spd, or Kn + Spd application. Data is the mean (±SE) of four replicates, and different letters represent a significant difference at *p* < 0.05.

**Figure 5:**
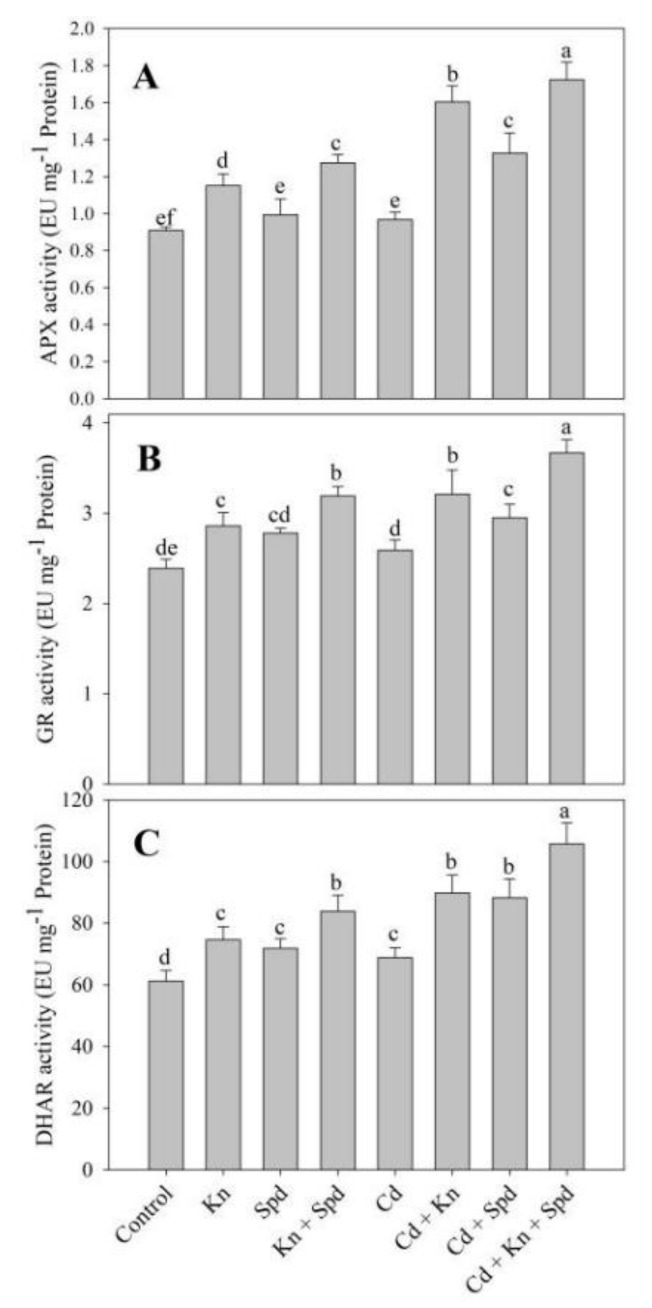
Effect of cadmium stress on activity of (**A**) ascorbate peroxidase, (**B**) glutathione reductase, and (**C**) dehydroascorbate reductase in *Vigna angularis* seedlings treated with exogenous Kn, Spd, or Kn + Spd application. Data is the mean (±SE) of four replicates, and different letters represent a significant difference at *p* < 0.05.

The activities of antioxidant enzymes assayed including SOD, CAT, APX, GR, and GST exhibited an increase due to the application of Kn and Spd, attaining maximal increments of 44.44%, 30%, 28%, 25%, and 35% due to Kn + Spd over control (Figure 4 and Figure 5). Relative to control, Cd stress increased the activity of SOD (18.23%), APX (5%), GR (7%), and GST (21%), while a decrease in CAT (21%) was observed. The application of Kn and Spd to Cd-stressed seedlings further increased the activities of antioxidant enzymes and attained a maximal increase of 41% for SOD, 29% for CAT, 43% for APX, 29% for GR, and 35% for GST in Cd + Kn + Spd-treated seedlings over Cd-stressed ones (Figure 4 and Figure 5).

**Figure 6:**
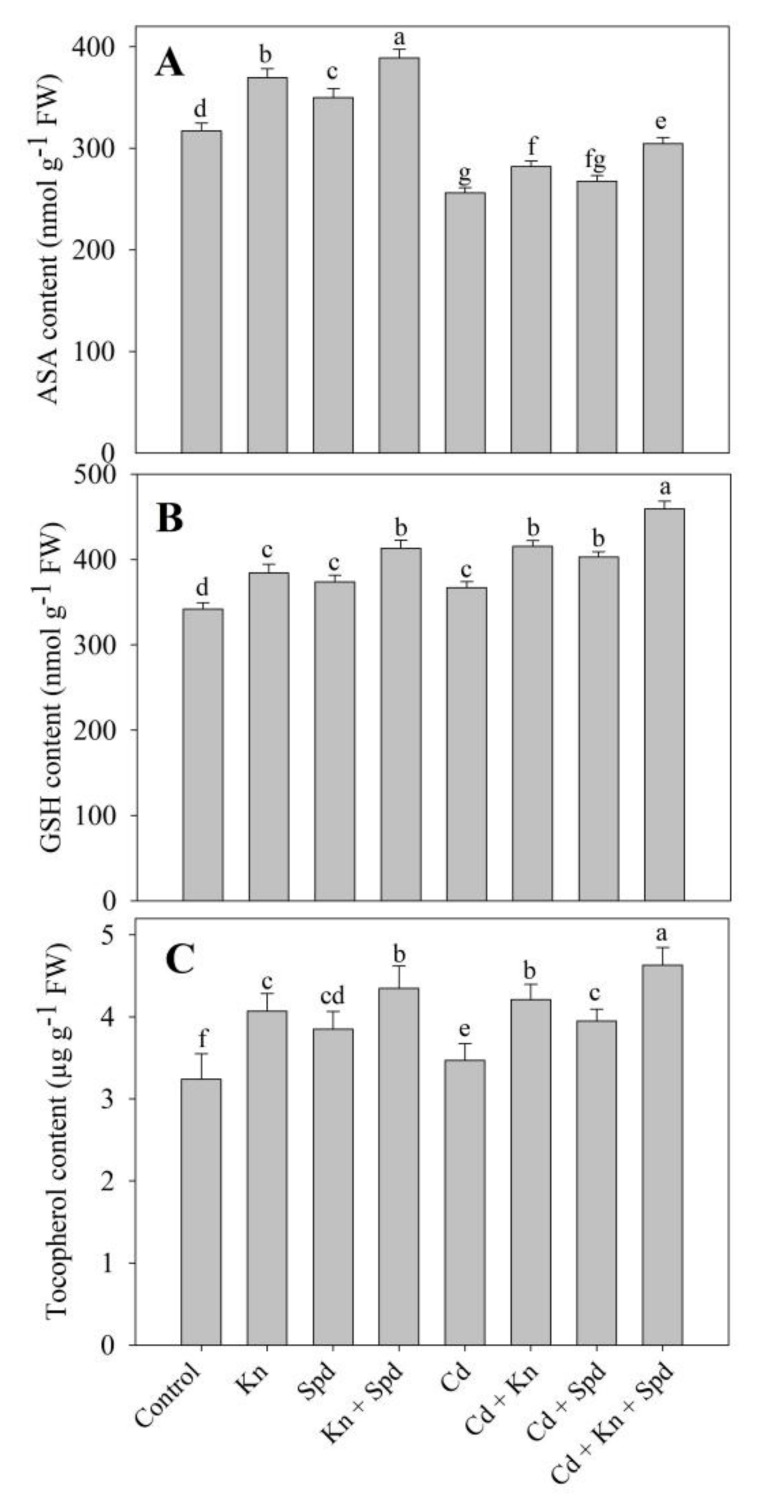
Effect of cadmium stress on content of (**A**) ascorbic acid, (**B**) reduced glutathione, and (**C**) tocopherol in *Vigna angularis* seedlings treated with exogenous Kn, Spd, or Kn + Spd application. Data is the mean (±SE) of four replicates, and different letters represent a significant difference at *p* < 0.05.

Relative to control, the content of AsA, GSH, and tocopherol increased due to the exogenous application of Kn and Spd. Under normal growth conditions, a percentage increase in AsA (18%), GSH (17%), and tocopherol (25%) was maximum in seedlings treated with Kn + Spd over control. Cd stress resulted in declined AsA (19%) and increased GSH (17%) and tocopherol (6%) content. The combined application of Kn + Spd maximally assuaged the Cd-mediated decline in AsA (15%) over the Cd-stressed plants and caused the further enhancement of 20% and 25% in GSH and tocopherol (Figure 6).

### 3.6. Effect of Kn and Spd on Phenol and Flavonoid Accumulation under Cd Stress

**Figure 7:**
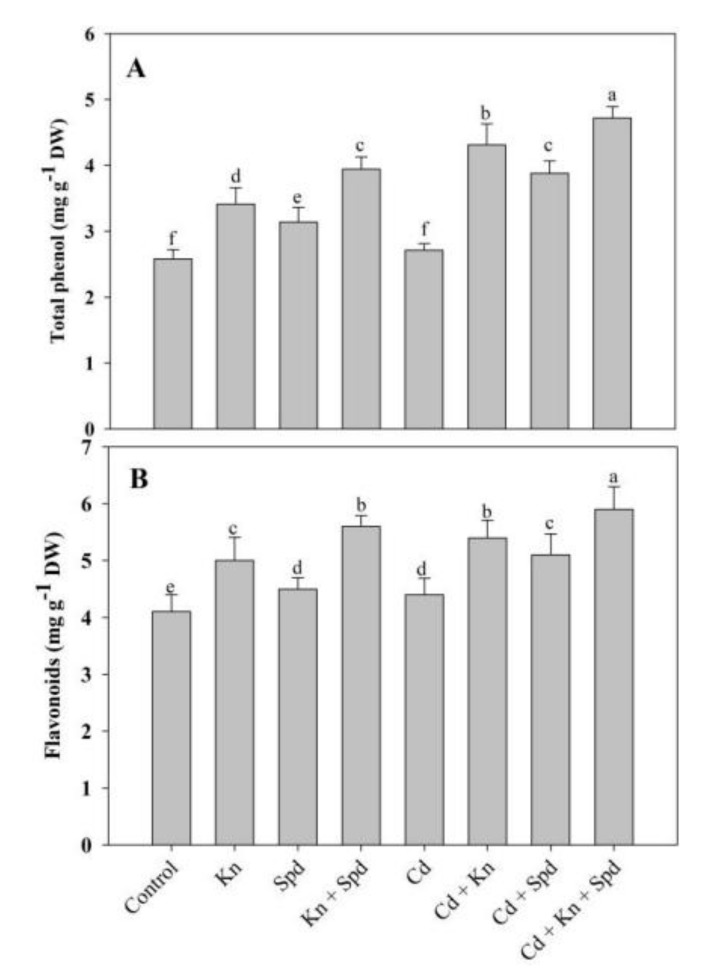
Effect of cadmium stress on content of (**A**) total phenol and (**B**) flavonoids in *Vigna angularis* seedlings treated with exogenous Kn, Spd, or Kn + Spd application. Data is the mean (±SE) of four replicates, and different letters represent significant differences at *p* < 0.05.

The application of Kn and/or Spd increased the accumulation of phenols and flavonoids significantly, attaining a maximal increase of 34% and 26% in seedlings treated with Kn + Spd over the control. Relative to control, Cd stress induced a slight accumulation of phenols (4%) and flavonoids (6%); however, the application of Kn or Spd individually further enhanced their accumulation. Relative to Cd-stressed plants, Cd + Kn + Spd-treated plants exhibited a further increase of 42% and 25% for phenols and flavonoids, respectively (Figure 7).

### 3.7. Activity of NR and N Content Increased Due to Kn and Spd Application

**Figure 8:**
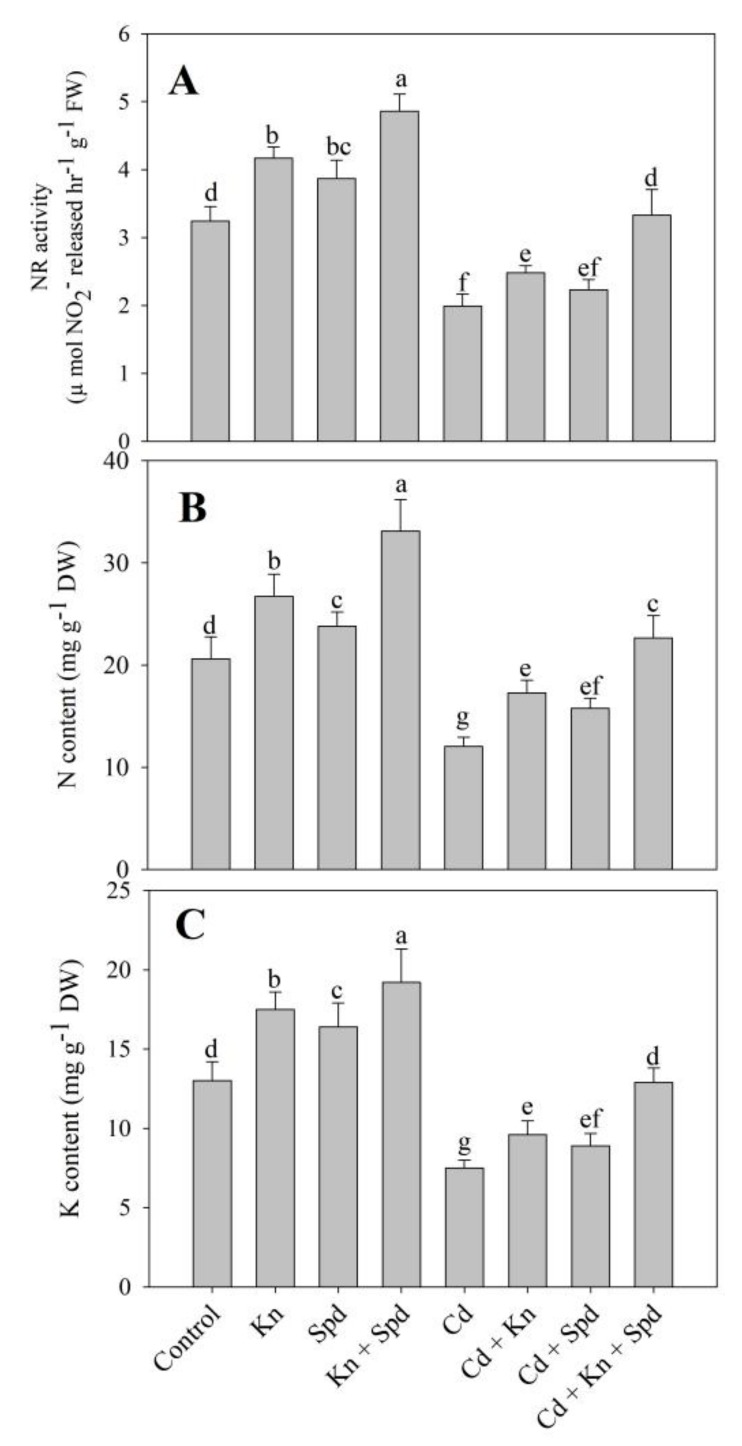
Effect of cadmium stress on activity of (**A**) nitrate reductase, and content of (**B**) nitrogen and (**C**) potassium in *Vigna angularis* seedlings treated with exogenous Kn, Spd, or Kn + Spd application. Data is the mean (±SE) of four replicates, and different letters represent significant differences at *p* < 0.05

Seedlings exposed to Cd stress exhibited declined activity of NR (38%) and N (41%) content over the control. The application of Kn, Spd, and Kn + Spd increased the activity of NR by 22%, 16%, and 33% respectively, causing an increase of 22%, 13%, and 37% respectively in N content. Such effects of the individual and combined application of Kn and Spd were maintained under Cd stress, also resulting in significant amelioration. Relative to Cd-stressed plants, a decline in the activity of NR and N content was mitigated maximally by 40% and 46% in Cd + Kn + Spd-treated plants (Figure 8A,B). Kn and Spd application also significantly increased the content of K over the control, while Cd stress declined K by 42% over the control. Relative to Cd-stressed plants, K content increased significantly due to Kn and Spd application with a maximal increase of 41% in Cd + Kn + Spd-treated seedlings (Figure 8C).

## 4. Discussion

Similar to other stresses, heavy metals considerably damage the normal growth and development of crop plants. Increasing heavy metal pollution results in a loss of productivity. Therefore, strategies have been developed to lessen the negative effects of these toxic metals by either (a) declining their uptake into plants or by (b) strengthening the tolerance mechanisms. In this connection, the use of phytohormones has emerged as an interesting alternative to assist crop plants to counteract the damaging effects of heavy metals. The present study investigated the beneficial effects of the individual and combined application of Kn and Spd in ameliorating the damaging effects of Cd stress in *Vigna angularis.* Reduced growth and biomass accumulation due to Cd stress has earlier been reported by Ahmad et al. [5] in tomato. Kn application has been reported to improve growth in *Pteris* exposed to arsenic stress [49]. It has been reported that Kn act synergistically with PAs to improve plant growth [50]. In the present study also, the combined application of Kn and Spd imparted maximal beneficial effects on the growth of adzuki beans, resulting in the considerable amelioration of the Cd-mediated decline. Cd declines plant growth and cellular proliferation by affecting the cellular division [51], while Kn improves cell division, therefore improving the growth and development [52]. Spd application further enhanced the beneficial effects of Kn on growth, thereby ameliorating the decline induced by Cd. Improved growth in Spd and Kn-treated seedlings was correlated with a reduced accumulation of Cd in them. An earlier application of Kn [7] and putrescine [53] individually has been reported to reduce the uptake of Cd significantly.

Increased growth following application of phytohormones results from their impact on photosynthesis. Cadmium stress declined the synthesis of chlorophyll and the rate of photosynthesis significantly; however, Kn and Spd application proved beneficial in mitigating the decline. Similar to our results, declined chlorophyll and photosynthesis due to Cd stress has been reported by Khan et al. [54] and Ahmad et al. [5]. Under stress, the chlorophyll degradation is accelerated due to the up-regulation of the degradative enzymes such as chlorophyllase, which is accompanied by the down-regulation of biosynthetic pathway [55]. Spd application has been reported to increase the synthesis of chlorophyll intermediates and decrease chlorophyllase activity [26]. In corroboration to our finding, earlier, Ahanger et al. [8] and Nahar et al. [53] have also reported the amelioration of salinity and a cadmium stress-induced decline in chlorophyll content due to the exogenous application of Kn and polyamine (putrescine). The increased synthesis of photosynthetic pigments and rate of photosynthesis is directly influenced by the significant impact on the uptake of key mineral ions and the regulation of stomatal characteristics. In the present study, the application of Kn and Spd was observed to improve N uptake and stomatal functioning. Increased N uptake contributes to greater Rubisco generation, while stomatal functioning maintains internal CO_2_ concentration and water, thereby leading to temperature maintenance [56]. Increased photosynthesis directly influences the growth and metabolite production, ultimately increasing the energy status [57]. The exogenous application of Kn has been reported to prevent the structural deformities in chloroplasts under metal stress [49]. Reports discussing the combined effects of Kn and Spd on the chlorophyll synthesis and photosynthesis are not available. In tomato exposed to salinity-alkalinity stress, Hu et al. [58] have demonstrated increased D1 protein synthesis and amelioration of the decline in photosynthesis due to the exogenous treatment of Spd. In addition, exogenous Spd application reduces chlorophyll degradation and enhances the stabilization of chloroplast structures [26].

The activity of nitrate reductase declined due to Cd stress, and the decline was significantly ameliorated by the exogenous application of Kn and/or Spd. Similar to our results, a reduced activity of nitrate reductase and N content have been reported in Cd-stressed wheat [54]. Nitrate reductase mediates the rate-limiting step in N metabolism, thereby regulating the key metabolic pathways including the amino acid and N-containing secondary metabolites [59]. Bashri et al. [60] and Khalil et al. [61] have also demonstrated increased nitrate reductase activity due to the application of cadaverine and Kn. Increased nitrate reductase activity results in improved N assimilation [61], thereby influencing the protein synthesis and stress tolerance [62]. In the present study, Kn and Spd mediated enhancement in the nitrate reductase, and N content may have directly regulated the synthesis of photosynthetic enzymes and other protective compounds. Furthermore, Kn and Spd application improved K uptake significantly under normal as well as Cd stress conditions. Increased K content directly influences plant growth through its involvement in the regulation of enzyme activity, osmolarity, and photosynthesis [9,63,64].

The application of Kn and Spd considerably declined the accumulation of ROS, including H_2_O_2_ and O_2_ under Cd stress. Earlier, Ahmad et al. [1] and Ahanger et al. [8] have also reported increased ROS accumulation in Cd and salinity-stressed plants. The earlier application of Kn [7] and putrescine [53] to Cd-stressed tomatoes and mungbeans have been reported to reduce the accumulation of H_2_O_2_ and O_2_^−^. However, their accumulation under the combined application of Kn and Spd has not been reported. Excess H_2_O_2_ and O_2_^−^ can hamper key cellular processes such as photosynthesis by reducing the stability of organelle structures. An excess accumulation of H_2_O_2_ and O_2_^−^ under stress leads to increased damage to proteins and lipids, thereby affecting their integrity and functioning, which was also apparent in the present study as increased lipid peroxidation and electrolyte leakage. Cd stress-mediated enhancement in the ROS accumulation results in the activation of lipoxygenase, which is an indicator of greater damage to lipids. In the present study, lipoxygenase activity was reduced by the exogenous application of Kn and Spd. Nahar et al. [53] have also reported reduced lipoxygenase activity due to putrescine application under Cd stress. Lipoxygenase generates unsaturated fatty acid hydroperoxide by adding molecular oxygen to polyunsaturated fatty acids and can also produce excess acyclic or cyclic compounds due to fatty acid oxidation [65]. Such increased membrane stability due to the combined application of Kn and Spd may be due to the maintenance of increased antioxidant activity in them.

Reduced oxidative damage in Kn and Spd-treated seedlings under normal as well as Cd-stressed conditions was ascribed to the significant up-regulation of the antioxidant system in them. The activities of antioxidant enzymes assayed including SOD, CAT, GST, and the components of the ascorbate–glutathione (AsA-GSH) cycle significantly increased due to Kn and Spd treatment. Different antioxidant enzymes have specific roles and share different locations within cells [66]. SOD is ubiquitous in the elimination of superoxide, while CAT scavenges hydrogen peroxide in cytosol. Hydrogen peroxide is also neutralized by the APX-GSH cycle with the concomitant generation of redox components including GSH, AsA, as well as NADPH, so that electron transport is maintained. Recently in *Anthurium*, de Moura et al. [67] have reported increased activities of antioxidant enzymes due to the foliar application of Kn, resulting in improved photosynthetic performance. Cadmium reduces the endogenous cytokinin synthesis and induces oxidative damage to lipids through the excessive generation of ROS, thereby reducing growth [68]. Singh and Prasad [7] have reported increased photosynthesis in cadmium-stressed *Solanum melongena* through the Kn mediated up-regulation of the antioxidant system and accumulation of non-protein thiols. In corroboration with our results earlier, Nahar et al. [53] and Li et al. [26] have also reported up-regulation of the antioxidant system due to the treatment of putrescine and Spd under Cd and salinity–alkalinity stress. The interaction of polyamines and cytokinin with other phytohormones such as indole cetic acid, giberellic acid, and nitric oxide have worked [53,69]; however, reports discussing the combined influence of Kn and Spd are not available. The increased functioning of the AsA-GSH cycle in Kn + Spd-treated plants may have contributed to greater protection to cellular organelles and pathways from the Cd-induced deleterious effects. Cd alters the redox homeostasis and thereby affects enzyme functioning, photosynthesis, and tolerance potential [2]. GSH and AsA form important components of the AsA-GSH cycle and are key to the functioning of its enzymatic components, including APX, DHAR, and GR. Increased GR activity leads to the maintenance of NADP concentration so that electron transport is not impeded and the generation of toxic radicals is prevented [8,70]. Metabolites including AsA and GSH are included in active radical scavenging within different cellular organelles [11], and a Kn + Spd-mediated increase in their accumulation may have further strengthened the tolerance against Cd. Tocopherol is another important antioxidant molecule that is found in thylakoid membranes, the chloroplast envelope, and plastoglobuli, and protects photosynthesis and growth by neutralizing the superoxide and hydroxyl radicals [71]. Increased tocopherol accumulation results from the altered gene expression of its degrading and recycling pathways [71]. Ye et al. [72] have demonstrated that the foliar application of tocopherol improves stress tolerance by enhancing the proline content. In the present study, Kn and Spd-mediated enhancement in tocopherol accumulation may have also improved other stress-responsive metabolites for the quick amelioration of oxidative effects of Cd.

In corroboration with our findings, increased osmolyte accumulation due to Cd stress has been reported by several researchers [1,5,54]. Reports discussing the role of Kn or Spd application in the improvement of osmolyte accumulation are available; however, their combined effect on the modulation of osmolyte accumulation under stress has not been worked. The increased accumulation of sugars, proline, and glycine betaine prevents the damaging effects of stresses by (a) maintaining tissue water content, (b) protecting enzyme activity and photosynthesis, and (c) scavenging ROS [11,73]. Increased glycine betaine [10] and proline [54] accumulation under salinity and Cd stress respectively have been reported to prevent oxidative effects on plant photosynthetic machinary, thereby imparting increased stress tolerance. A greater accumulation of compatible osmolytes including sugars and proline positively influences the functioning of key enzymes [59], and such results were evident in our study also. Increased osmolyte accumulation due to Kn + Spd application may have contributed to free radical elimination, thereby preventing photoinhibition and membrane damage. The increased accumulation of proline results from the up-regulation of the biosynthesis pathway [54], and Kn and/or Spd may have influenced the pathway; however, future research investigations are needed. Increased stress tolerance due to osmolyte accumulation following the application of Kn [8] and Spd [74] has been reported.

Phenols and flavonoids include secondary metabolites sharing key functions in plant growth maintenance under stressful conditions. In the present study, the beneficial impact of exogenously applied Kn and Spd individually or combined was evident, thereby strengthening the ROS scavenging systems besides their key beneficial effects on growth [11]. The increased accumulation of phenols and flavonoids has been also reported by Ibrahim et al. [75] in *Gynura procumbens*. The increased accumulation of secondary metabolites has been reported to result in improved tolerance to toxic metals [76], and in the present study, Kn + Spd-mediated enhancement in the accumulation of secondary metabolites may have significantly contributed to avoid the deleterious effects of Cd. Secondary metabolites mediate stress signals for the elicitation of a better stress response [77]. Polyamines conjugate with phenols including ferulic acid and caffeic acid, resulting in their improved stability and translocation [78]. The increased accumulation of secondary metabolites results from the up-regulation of the activities of enzymes involved in their biosynthesis [9,63,75]. Flavonoids and phenols scavenge ROS including superoxide and hydrogen peroxide, thereby protecting major cellular structures and their functioning [11,79].

## 5. Conclusions

Conclusively, exogenous Kn and Spd individually or combined improved the growth of *Vigna angularis* by improving chlorophyll synthesis, photosynthesis, and enzyme activity. The impact was obvious due to their combined applications. Kn and Spd application proved to be significant in mitigating the oxidative effects of Cd stress on membranes by reducing the excess generation of ROS. Improved Cd tolerance due to the combined application of Kn and Spd was due to the significant up-regulation of the antioxidant system and osmolyte accumulation, and declined Cd accumulation justifies their synergistic interaction. However, further studies are required to strengthen the knowledge about the actual mechanisms involved.

## Figures and Tables

**Table 1:** Effect of cadmium stress on total chlorophyll (mg g^−1^ FW), carotenoids (mg g^−1^ FW), photosynthesis (μmol (CO_2_) m^−2^ s^−1^), transpiration rate (mol (H_2_O) m^−2^ s^−1^), intercellular CO_2_ (μmol(CO_2_) mol^−1^), RWC (percent), plant height (cm), plant dry weight (g plant^−1^), shoot and root cadmium (μmol g^−1^ DW) content treated with Kn, Spd, and Kn + Spd. Data is mean (±SE) of four replicates and different letters (a–f) represent significant difference at *p* < 0.05.

Parameters	Control	Kn	Spd	Kn + Spd	Cd	Cd + Kn	Cd + Spd	Cd + Kn + Spd
Total chlorophyll	1.13 ± 0.15 d	1.41 ± 0.10 b	1.23 ± 0.05 c	1.58 ± 0.03 a	0.772 ± 0.031 f	0.9306 ± 0.082 e	0.9172 ± 0.08 e	1.166 ± 0.16 cd
Carotenoids	0.4072 ± 0.01 c	0.5083 ± 0.01 b	0.4985 ± 0.02 b	0.5822 ± 0.015 a	0.2539 ± 0.01 f	0.3311 ± 0.016 d	0.3096 ± 0.01 e	0.3961 ± 0.02 c
Photosynthesis (Pn)	15.89 ± 1.0 c	18.28 ± 1.3 b	17.63 ± 1.1 b	23.70 ± 1.9 a	8.86 ± 0.7 f	11.03 ± 0.45 d	9.76 ± 0.51 e	16.2 ± 1.0 c
Transpiration rate	3.26 ± 0.05 c	3.96 ± 0.20 b	3.86 ± 0.15 b	4.20 ± 0.26 a	2.09 ± 0.30 f	2.93 ± 0.14 d	2.87 ± 0.20 de	3.26 ± 0.25 c
Intercellular CO_2_	238.3 ± 7.6 d	274.3 ± 6.6 b	268.0 ± 6.5 b	301.6 ± 9.0 a	160.6 ± 5.1 f	234.3 ± 3.5 d	228.6 ± 2.5 de	255.3 ± 4.0 c
RWC	81.63 ± 4.3 bc	86.88 ± 4.4 b	85.38 ± 5.5 b	90.80 ± 5.9 a	56.88 ± 3.2 f	69.67 ± 4.4 e	66.88 ± 4.3 e	73.66 ± 4.8 d
Plant height	19.6 ± 2.1 d	25.5 ± 2.1 b	22.30 ± 1.4 c	27.8 ± 2.1 a	13.4 ± 0.55 f	16.8 ± 0.71 e	15.7 ± 0.66 e	20.1 ±1.21 d
Plant dry weight	2.2 ± 0.11 d	3.4 ± 0.28 ab	2.98 ± 0.21 c	3.90 ± 0.24 a	1.43 ± 0.09 f	1.90 ± 0.11 e	1.70 ± 0.14 e	2.30 ± 0.24 d
Shoot Cd	nd	nd	nd	nd	55.23 ± 4.1 a	33.61 ± 3.0 b	35.23 ± 2.9 b	21.03 ± 2.3 c
Root Cd	nd	nd	nd	nd	107.26 ± 6.1 a	85.63 ± 3.6 b	77.96 ± 3.8 b	67.23 ± 3.2 c
